# Environmental Persistence of Monkeypox Virus on Surfaces in Household of Person with Travel-Associated Infection, Dallas, Texas, USA, 2021

**DOI:** 10.3201/eid2810.221047

**Published:** 2022-10

**Authors:** Clint N. Morgan, Florence Whitehill, Jeffrey B. Doty, Joann Schulte, Audrey Matheny, Joey Stringer, Lisa J. Delaney, Richard Esparza, Agam K. Rao, Andrea M. McCollum

**Affiliations:** Centers for Disease Control and Prevention, Atlanta, Georgia, USA (C.N. Morgan, F. Whitehill, J.B. Doty, A. Matheny, L.J. Delaney, A. K. Rao, A.M. McCollum; Dallas County Health and Human Services, Dallas, Texas, USA (J. Schulte, J. Stringer, R. Esparza)

**Keywords:** monkeypox virus, MXPV, orthopoxvirus, viruses, monkeypox, environmental persistence, fomites, virus diseases, virus shedding, travel-associated infection, disease transmission, surfaces, household, infection, zoonoses, Dallas, Texas, United States

## Abstract

In July 2021, we conducted environmental sampling at the residence of a person in Dallas, Texas, USA, who had travel-associated human West African monkeypox virus (MPXV-WA). Targeted environmental swab sampling was conducted 15 days after the person who had monkeypox left the household. Results indicate extensive MPXV-WA DNA contamination, and viable virus from 7 samples was successfully isolated in cell culture. There was no statistical difference (p = 0.94) between MPXV-WA PCR positivity of porous (9/10, 90%) vs. nonporous (19/21, 90.5%) surfaces, but there was a significant difference (p<0.01) between viable virus detected in cultures of porous (6/10, 60%) vs. nonporous (1/21, 5%) surfaces. These findings indicate that porous surfaces (e.g., bedding, clothing) may pose more of a MPXV exposure risk than nonporous surfaces (e.g., metal, plastic). Viable MPXV was detected on household surfaces after at least 15 days. However, low titers (<10^2^ PFU) indicate a limited potential for indirect transmission.

Monkeypox, a zoonotic infectious disease caused by monkeypox virus (MPXV; genus *Orthopoxvirus* [OPXV]), is endemic to West and Central Africa. After its discovery in 1958, the virus had not been reported in humans outside its endemic range until 2003, when a shipment of MPXV-infected small mammals was transported from Ghana to the United States, causing secondary animal and human infections (*1*). Genomic sequencing of isolates of MPXV across its endemic range indicates the existence of 2 clades: West African (WA) and Congo Basin; WA MPXV has a lower mortality rate (*2*).

Transmission is known to occur by direct contact with infectious lesion material or bodily fluids of an infected human or animal or by inhalation of respiratory secretions during prolonged, face-to-face contact (*3*,*4*). In addition, transmission might occur by direct contact with objects or materials contaminated with MPXV, although documented occurrences are rare (*5*,*6*). Poxvirus lesions, their exudates (vesicular or pustular fluid), and crusts contain viable virus (*3*,*4*). Poxvirus virions within lesion material shed during infection are known to be more resistant to desiccation than for other enveloped viruses (e.g., influenza viruses, rubella virus) because the virions are tightly bound with the fibrin matrices of the scab/crust material (*7*,*8*). This feature can lead to long-term environmental persistence of OPXVs.

Studies with variola (causative agent of smallpox) and vaccinia viruses demonstrated that if contaminated material is maintained in an environment that has low humidity, low temperature, and remains protected from UV radiation, the viral particles can remain viable for months to years (*9*,*10*). One study demonstrated the extreme longevity of variola virus in lesion scabs stored within an envelope in a cupboard; the virus remained viable for 13 years until the sample was used to completion (*11*). This longevity is striking; however, the infectivity of variola scabs alone is believed to be low based on epidemiologic and laboratory data (*11*–*14*). Vesicular fluid from OPXV lesions and other secretions generally have lower persistence in some laboratory studies than scabs (*15*,*16*). Outside of insights gained from other OPXVs, the longevity and environmental persistence of MPXV is largely unknown, including within a household environment. Considering the increasing frequency of monkeypox cases being exported from disease-endemic areas (*17*–*20*), and the concern for secondary infections among household contacts, there is a need for more specific information on transmission risks due to contaminated fomites in the household.

On July 16, 2021, the US Centers for Disease Control and Prevention (CDC) confirmed a WA MPXV infection in a man (US resident) who had recently traveled from Lagos, Nigeria, to Dallas, Texas, USA. This case was the first MPXV infection reported in the United States since the 2003 outbreak, prompting an immediate response and investigation. The person who had monkeypox arrived in Dallas on July 9 and stayed in a household, a 1-bedroom residence that had no other occupants, for 4 days before coming to the hospital for treatment (*17*). During these 4 days, the man was in the household and had a disseminated purulent rash (*17*). We conducted environmental sampling within the residence and assessed the viral load and viability of virus present on commonly used surfaces and objects within the household.

## Methods

### Site Information

Environmental sampling took place in July 2021, 15 days after the person who had monkeypox departed the residence for the hospital. The person was interviewed by CDC, Dallas County Health and Human Services, and hospital officials regarding condition of residence, activities within the household before hospital admittance, and locations of potentially soiled materials and high-touch objects. We recorded notes on specific location, soiled condition of surfaces, and light exposure for each sample collected.

### Personal Protective Equipment

Personnel performing the household environmental sampling were vaccinated with ACAM2000 (Sanofi Pasteur, https://www.sanofi.com), in accordance with recommendations for personnel at risk for occupational exposure to OPXVs (*21*). Before entering the residence, the sampling team donned personal protective equipment, including Tyvek (DuPont, https://www.dupont.com) coverall with hood, inner and outer nitrile gloves, fit-tested N95 filtering facepiece respirator, and face shield. Because of the public setting, discretion was used when entering the residence, avoiding areas of high visibility, and personal protective equipment was donned at the exterior entrance immediately before entering.

### Sample Collection

The sampling team collected swab samples from high-use objects and environmental surfaces within the household that would probably have had direct contact with the person who had monkeypox and objects that appeared visibly soiled. Individually wrapped sterile cotton-tipped applicator swabs (Puritan, https://www.puritanmedproducts.com) were removed from their packaging and prewetted by inserting into a corresponding labeled 2-mL cryotube (Sarstedt, https://www.sarstedt.com) filled with 300 μL of phosphate-buffered saline (PBS; pH 7.4). The swab was then immediately applied to the environmental surface and swabbed vigorously for 10 seconds while rotating the swab to ensure all sides of the swab contact the environmental surface. On all surfaces and objects, an approximate area of 2 in^2^ was swabbed, and if soiled, the soiled area was targeted. Aseptic techniques were used, and outer gloves were changed between samples or if soiled. There is not a fully validated environmental sampling method for OPXVs, so the sampling procedure was adapted from similar studies with vaccinia virus and SARS-CoV-2 (*22*,*23*).

### Sample Processing and PCR Testing

We stored all swab samples and shipped them in sealed 2-mL cryotubes containing ≈300 μL PBS and kept refrigerated (2°C–4°C) until processing. We transferred swabs and the 300 μL of sterile PBS within the tube to the swab extraction tube system (SETS; Roche, https://www.roche.com). We then centrifuged SETS tubes at 6,000 rpm for 1 min to collect the elute, after which we discarded inner SETS tubes and swabs. We aliquoted 100 μL of swab eluate and used it for DNA extraction; the remaining eluate was kept for viral titration. We extracted DNA from all samples by using the EZ1 DNA Tissue Kit and Biorobot System (QIAGEN, https://www.qiagen.com). We screened all samples for MPXV DNA by real-time PCR using the WA MPXV-specific assay (*24*) on the VIIA7 Real-Time PCR System (Applied Biosystems, https://www.thermofisher.com).

### Virus Isolation and Titration

We put swab eluate from all samples into cell culture to attempt virus isolation and assess presence of viable virus. We added a 100-μL aliquot of swab eluate to BSC-40 cell monolayers (African green monkey kidney cell line) in T-25 cell culture flasks and incubated at 35.5°C in an atmosphere of 6% CO_2_, in Roswell Park Memorial Institute medium as described (*25*). We observed infected T-25 flasks daily for cytopathic effect (CPE), incubated for a maximum of 14 days or until positive. We harvested material from flasks if considered positive for viable virus (successful viral isolation attempt), when ≈100% of monolayer showed CPE activity. For an additional confirmation of the successful virus isolation attempt, we tested an aliquot of the harvested flasks material by using PCR.

Swabs collected from environmental surfaces might result in bacterial or fungal contamination during virus isolation attempts. To help mitigate bacterial or fungal overgrowth in T-25 flasks, we supplemented cell culture medium with penicillin/streptomycin, amphotericin B, and gentamicin. If either bacterial or fungal contamination was identified, 4 cycles of removing medium and adding fresh medium to wash monolayers was conducted as often as necessary to prevent overgrowth.

After PCR and attempt at virus isolation, samples from which virus was isolated were evaluated by using viral titration accords to methods described (*26*). Because of low sample volume, we added 50 μL of PBS diluent to 100 μL of swab eluate from each positive sample and serially diluted them in 2% Roswell Park Memorial Institute medium, and we added 650 μL of each dilution to 6-well plates in duplicate on BSC-40 cell monolayers. We incubated plates at 35.5°C in an atmosphere of 6% CO_2._ After a 72-hour incubation period, we inactivated and stained plates with 2× formalinized crystal violet stain and then enumerated plaques. Titers are expressed as PFU/mL.

### Statistical Analyses

We performed statistical analyses to compare PCR positivity, average cycle threshold (Ct) value, and viral culture positivity of environmental swab samples from porous and nonporous surface types collected in the household. We conducted statistical analyses by using OpenEpi version 3.01 (https://www.OpenEpi.com).

## Results

### Site Information

We conducted interviews with the person who had monkeypox detailed limited activities within the household during the 4-day period between returning from travel and admittance to the hospital. The person reported sleeping/resting on the bed in the bedroom, spending prolonged time on 2 couches in the living room, showering, and retrieving food from the kitchen refrigerator. The person detailed the location of clothing items worn during travel, as well as other clothing worn while in the household. The heating, ventilation, and air conditioning system of the residence was turned off when the person left for the hospital. The exact environmental conditions within the residence for 15 days are unknown, although it is probable they were nearing the external environmental conditions; the mean temperature was 86°F (range 73°F–101°F) and mean relative humidity 63% (range 49%–76%) (*27*). Within the residence, we collected 31 environmental swab samples. Of these 31 samples, 10 (32%) were from items and surfaces made of porous material, such as cloth and paper, and 21 (68%) were from nonporous material, such as sealed wood and plastic (Table 1).

**Table 1:** Objects and surfaces swabbed during environmental sampling of residence of a patients who had monkeypox, Texas, USA, 2021*

Object/surface	Room, specific location	Sample from visibly soiled surface	Sample exposed to UV	Surface type	Mean cycle threshold	Viral culture result, PFU/mL
Paper towels	Bedroom, on bed	Yes	Low	Porous	16.1	<1 × 10^2^
Underwear	Bedroom, on bed	Yes	No	Porous	17.9	<1 × 10^2^
Underwear	Bedroom, on bed	Yes	No	Porous	19.3	3.2 × 10^2^
Blanket	Living room, on couch	Yes	Low	Porous	20.3	<1 × 10^2^
Towel	Living room, on couch	Yes	Low	Porous	20.3	<1 × 10^2^
Disinfectant wipes	Bedroom, bedside table	No	Low	Porous	21.9	‒
Towel	Bedroom, on bed	Yes	Low	Porous	22.3	‒
Mattress cover	Bedroom closet, in hamper	Yes	No	Porous	23.1	<1 × 10^2^
Towel	Bedroom, near bathroom	Yes	Low	Porous	36.7	‒
Underwear	Bedroom, near bathroom	Yes	No	Porous	UND	‒
Coffee table top	Living room, edge near couch	No	Low	Nonporous	21.6	<1 × 10^2^
Bedside table	Bedroom, at bedside	No	Low	Nonporous	21.7	‒
Sink knobs	Bathroom, at sink near entry	No	Low	Nonporous	22.3	‒
Bathtub drain	Bathroom, in bathtub	No	Low	Nonporous	24.4	‒
Toilet seat	Bathroom, on toilet	Yes	Low	Nonporous	24.7	‒
Light switch	Bathroom, at entrance above sink	Yes	Low	Nonporous	25.0	‒
Closet doorknob	Outer knob of closet door in bedroom	No	Low	Nonporous	25.2	‒
Dresser top	Bedroom, near entry	No	Low	Nonporous	25.9	‒
Refrigerator handle	Kitchen, on refrigerator door	Yes	Low	Nonporous	26.7	‒
Toilet handle	Bathroom, on toilet	No	Low	Nonporous	26.7	‒
Bathtub faucet	Bathroom, in bathtub/shower unit	No	Low	Nonporous	26.9	‒
Doorknob	Inner knob of bathroom door	No	Low	Nonporous	27.3	‒
Freezer handle	Kitchen, on freezer door	No	Low	Nonporous	28.2	‒
Cell phone	Living room, on coffee table	No	Low	Nonporous	28.3	‒
Light switch	Bedroom, at entrance above dresser	No	Low	Nonporous	33.3	‒
Light switch	Kitchen, on wall	No	Low	Nonporous	33.5	‒
Light switch	Living room, near front door	No	Low	Nonporous	33.9	‒
Power strip button	Bedroom, on floor along bed	No	Low	Nonporous	34.2	‒
Television remote	Living room, on TV stand	No	Low	Nonporous	35.7	‒
Microwave handle	Kitchen, on microwave	No	Low	Nonporous	UND	‒
Closet light switch	Bedroom, next to closet door	No	Low	Nonporous	UND	‒

*UND, undetermined (below detectable limit); ‒, negative.

All windows were covered with closed blinds, enabling limited sunlight into the household. Sunlight (UV) exposure is denoted as no UV, indicating surface sampled was completed covered or kept in dark room, or low UV, indicating surface was uncovered and exposed to the low ambient light environment of the household (Table 1).

### PCR Testing

Overall, 27 (87%) samples amplified MPXV-WA DNA, and the mean cycle threshold (Ct) value was 25.83 (range 16.14–36.74). Swabs collected from porous materials were 90% (9/10) PCR positive, and those collected from nonporous materials were 90.5% (19/21) PCR positive (p = 0.94) (Table 2). Porous materials had higher detectable levels of viral DNA (Ct 21.98) than did nonporous materials (Ct 27.65) (p<0.01) (Table 2). Among the PCR-positive swabs, detectable levels of viral DNA in each room within the household was, in order of highest to lowest: closet (Ct 23.08, n = 1); bedroom (Ct 24.96, n = 13); bathroom (Ct 25.33, n = 7); living room (Ct  26.66, n = 6); and kitchen (Ct 29.44, n = 4) (Table 1). Cell culture isolates considered positive were also tested by using PCR, and all were positive (Ct range 14.2‒16.0).

**Table 2:** Characteristics of environmental swab samples from porous and non-porous surface types collected in household of a patient who had confirmed monkeypox, Texas, USA, 2021

Surface type	No. samples collected	Positive by PCR, no. (%)	p value*	Average cycle threshold (SD)	p value†	Viable virus cultured, no. (%)	p value*
Porous	10	9 (90)	0.94	22.0 (6.0)	<0.01	6 (60)	<0.01
Nonporous	21	19 (90)		27.7 (4.4)		1 (5)	

*By mid-p exact test. †By 2-sample independent t-test.

### Virus Isolation and Titration

Viral isolation was attempted from all 31 swab samples, and 7 (23%) contained viable virus (Table 1). Virus isolation was successful with 23% (3/11) of swabs from the bedroom, 50% (3/6) from the living room, and 1 sample collected from a used mattress cover in the closet. Overall, 60% (6/10) of porous materials contained viable virus, and only 5% (1/21) of nonporous materials contained viable virus (p<0.01) (Table 2). The appearance of the first signs of CPE in the successful virus isolation attempts ranged from 2 to 8 (mean 5) days postinfection. We harvested material from flasks when CPE affected 100% of the monolayer at 6–12 (mean 9) days postinfection. We observed limited bacterial or fungal contamination in this study; only 1 sample (bathtub faucet) required >3 monolayer washes to curtail bacterial overgrowth.

Of the 7 culture-positive swab specimens, 6 were below the detectable limit (2.1 × 10^2^ PFU) of the titration assay (titers <1 × 10^2^ PFU/mL). Only sample TX-23 had a quantifiable titer of 3.2 × 10^2^ PFU/mL.

## Discussion

In this real-world setting, targeted sampling of high-use surfaces and objects was effective at detecting MPXV DNA and viable virus. MPXV DNA was found throughout the household, indicating extensive spread of viral material, probably a result of the man having an extensive purulent rash develop. Our study demonstrated the ability of MPXV to persist in a household environment for at least 15 days. Previous studies with vaccinia and variola viruses demonstrate the capability of OPXVs to persist in the environment much longer than 15 days (months or years), so potentially the virus sampled in this household could have remained viable for a longer period (*10*,*15*,*16*). A similar investigation had been conducted with a household case of eczema vaccinatum (vaccinia virus), in which household contamination was a concern because of extensive rash (*22*). That study reported less extensive viral DNA dissemination around the household than what we detected and viable virus from 1 cloth item and 2 nonporous items at 10 days after removal of an infected person (*22*).

In a household environment, myriad physical, chemical, and biologic factors could affect the persistence of OPXVs (Appendix Table). Most viruses exhibit greater persistence on nonporous surfaces; however, OPXVs typically have higher persistence on porous surfaces and show a high resistance to drying (*28*,*29*). In this study, we detected viable virus on multiple substrate types, including cellulose fiber sheets (paper towels), cotton and cotton/synthetic blended fabrics (towel, blanket, underwear, mattress cover), and sealed wood veneer (coffee tabletop). Items considered porous had increased detectable levels of viral DNA, and viable virus was only detected from 1 nonporous surface.

Despite the hardiness of OPXVs, it is probable there was some degree of viral decay of MPXV before sampling. This decay could have occurred either because of environmental and physical properties of the residence and surface types or contact with a disinfectant or soap. For example, 4 culture-negative samples had MPXV-WA Ct values higher than that of the mattress cover, which was culture positive. One of these samples was collected from dried disinfectant wipes (Clorox, https://www.thecloroxcompany.com), which probably successfully inactivated the virus. In addition, no viable virus was detected in the 7 samples collected from the bathroom, even though the person who had monkeypox spent much time in this room; this result could also be the result of contact with disinfectant or soap. The bathroom samples, if not inactivated by contact with disinfectants, could possibly have been culture negative because of the nonporous surfaces and higher humidity that would occur in the bathroom during showering or sink use. It is useful in studies assessing environmental persistence or contamination of MPXV to attempt viral isolation from PCR-positive samples. In environmental sampling studies, low Ct values should not be used as a proxy for viable virus because many factors and environmental conditions might lead to complete viral decay on contaminated surfaces.

Interviews with the person who had monkeypox in this study showed that most of the time in the residence was spent lying down or resting. As a probable result, it was only items on or near the bed and couches from which viable virus was detected. The results of this study demonstrate the need to take special precautions when handling bedding, clothing, or towels of a person who has monkeypox. When there are other occupants in a household, or visitors, persons who have monkeypox should not share bedding, clothing, towels, or sleeping and living spaces.

During 2018, a healthcare worker in the United Kingdom became infected, probably as a result of handling the bedding of a person who had monkeypox without using respiratory protection (*30*). The persistence of the virus in the environment depends largely on the viral load and type of infectious material initially deposited, environmental conditions, and physical/chemical properties of the contaminated objects. Furthermore, if fomites in the environment have sufficient amounts of viable virus, the capability of these materials to cause secondary infection is probably dependent on route of exposure, including opportunistic contact or transfer to mucous membranes, or preexisting immunity.

The only culture-positive swab sample with sufficient viral load to reach the detectable limits of our titration assay was from an article of clothing that had prolonged, direct contact with purulent lesions; the titer was 3.2 × 10^2^ PFU/mL (detection limit 2.1 × 10^2^ PFU/mL). There are few data on the infectious dose necessary to cause infection in humans. However, these data can be inferred from laboratory challenge studies with the prairie dog animal model. Virus titers of 10^4^ and 10^3^ PFU in most cases cause infection, and in 1 study, 1 of 4 prairie dogs infected with 6 × 10^2^ PFU MPXV-WA became infected and showed development of disseminated lesions (*26*,*31*). This result might indicate that in otherwise healthy persons, a viral load on the order of 10^2^ PFU is the lower threshold for infection, and at these levels the innate immune system can potentially clear the virus.

It is unknown how long the culture-positive materials in this study would remain viable with MPXV because viral titers will decrease over time until undetectable by viral culture. Subsequently, as viral titers decrease, infectivity (capacity to cause secondary infection) would also decrease. In comparable studies of OPXV persistence (Appendix Table), under slightly lower heat and humidity conditions, the maximum duration that virus remained detectable on fabrics ranged from 28 to 70 days (*16*,*32*). Considering the low titers observed (<3.2 × 10^2^ PFU/mL) and the high heat and moderate humidity environment, it is probable that maximum persistence of viable virus on the items sampled would fall into a similar range.

This study was conducted alongside a public health response, and priorities were identifying potential high transmission risk areas and objects and confirming presence/absence of viable virus. These results are merely representative of the conditions of each specific item at 1 point in time and not representative of the total potential for fomite transmission within the household from items not sampled. A more robust sampling method would be recommended for future studies, including multiple sampling time points and recording the environmental conditions in the household over time. Household disinfection is recommended for any household occupied by a person confirmed to have monkeypox. A disinfectant registered with the Environmental Protection Agency should be used, such as a disinfectant that has an emerging viral pathogens claim, in accordance with the manufacturer’s instructions (*33*). Specific recommendations for household disinfection can be found on the CDC monkeypox web page (*34*).

After this case in July 2021, two additional travel-associated cases were detected in the United States and the United Kingdom (*19*,*20*), and in May 2022, an unprecedented number of monkeypox cases were identified from multiple clusters worldwide (*35*,*36*). Current outbreaks of human monkeypox cases in endemic and nonendemic regions, and the increasing frequency of which travel-associated cases are occurring, necessitate a further need to understand transmission dynamics within a household setting. Since the 2022 outbreak began, an additional study on monkeypox contamination within a household has been published and detected comparable levels of viral contamination despite differences in sampling and processing methods (*37*). In that study, samples were collected 3 days after the patient was last in the residence, and similar to our study, virus was isolated mostly from porous surfaces. However, the authors reported successful MPXV isolation from 40% (2/5) of nonporous surfaces sampled from which isolation was attempted (door handle and handheld electronic device). Because we report isolation of MPXV from just 5% (1/21) of nonporous surface sampled after 15 days, this finding potentially indicates that MPXV decay occurs more rapidly on nonporous than porous surfaces, as was reported for other OPXVs (*28*,*29*).

Future studies should develop a validated environmental sampling protocol for OPXVs and explore additional sample collection methods, comparing multiple applicator types and transport media because they might affect viral recovery during processing and overall viral yield (*38*,*39*). Additional studies on household transmission risks should be conducted to inform public health responses and cleaning and disinfection protocols, and to provide specific recommendations to at-risk communities and persons. Furthermore, documenting the risk potential of object-specific fomites and materials within a household will inform recommendations for infection prevention strategies and cleaning and disinfecting efforts.

Appendix Additional information on environmental persistence of monkeypox virus on surfaces in household of person with travel-associated infection, Dallas, Texas, USA, 2021.
